# PtrIAA12-PtrARF8 Complex Regulates the Expression of *PtrSAUR17* to Control the Growth of Roots in *Poncirus trifoliata*

**DOI:** 10.3390/plants14182875

**Published:** 2025-09-16

**Authors:** Xiaoli Wang, Manman Zhang, Xiaoya Li, Saihang Zheng, Fusheng Wang, Shiping Zhu, Xiaochun Zhao

**Affiliations:** 1 Citrus Research Institute, Southwest University/Chinese Academy of Agricultural Sciences, Beibei, Chongqing 400712, China; 2 National Citrus Engineering Research Center, Beibei, Chongqing 400712, China; 3 Yibin Academy of Southwest University, Southwest University, Yibin 644005, China

**Keywords:** citrus, rootstock, AUX/IAA-ARF interaction, PtrSAUR17, root development

## Abstract

The root system is an important determinant affecting the growth, adaptivity and stress resistance of citrus plants. Currently, the genetic regulatory network underlying root growth and development in citrus remains largely unknown. We report that a PtrAUX/IAA-ARF complex mediates the growth and development of roots in citrus through regulating the transcription of *PtrSAUR*. The auxin signaling pathway plays an essential role in regulating the growth and development of roots. In this study, we found that in citrus *Poncirus trifoliata*, *PtrIAA12*, encoding a canonical Aux/IAA protein, was highly expressed in the meristem and elongation zone of the root. Functional characterization showed that overexpression and silence of *PtrIAA12* significantly enhanced and suppressed the elongation of primary roots, respectively. Further analysis revealed that PtrIAA12 could interact with some members of *PtrARFs*, of which, PtrARF8 was identified to be the transcriptional factor of *PtrSAUR17*. Investigation of *PtrSAUR17* transgenic plants verified that *PtrSAUR17* is a key gene regulating the growth of roots in citrus. In conclusion, PtrIAA12 and PtrARF8 are the key members of the AUX/IAA-ARF complex in citrus controlling the growth and development of roots through regulating the transcription of *PtrSAUR17*.

## 1. Introduction

Grafting is the most important technique for the propagation of citrus. Rootstock plays an important role in the growth, development and biotic and abiotic stress resistance of citrus plants. The performance of rootstocks depends on the root system. A well-structured root system would enable citrus plants to access more sources of water and nutrients, while also providing them with better resistance to adverse environmental conditions. Therefore, understanding the development of citrus roots is of great importance to enhance the growth, development and stress resistance of citrus plants.

Root development is a complex process, regulated by several plant hormones, including auxin (IAA), cytokinin (CK), abscisic acid (ABA), brassinosteroid (BR), salicylic acid (SA), gibberellin (GA), etc. [1,2,3]. In fact, the crosstalk of plant hormones plays a vital role in the spatial and temporal coordination of root development [4]. Among those phytohormones, auxin is a central regulator of root growth and development; the biosynthesis, transport, distribution and signaling of auxin integrate with other hormones to regulate root architecture [3,5]. Auxin can not only regulate almost every facet of root development, including the primary root and lateral root, but also affect the gravitropic setpoint angle [5]. Modifying auxin synthesis, transport and signaling results in differences in auxin distribution at the organ and cellular level, which in turn alter growth rates, growth directions (tropisms), and organ initiation, such as root growth and development [6,7,8].

Canonical auxin signaling is mainly made up of TRANSPORT INHIBITOR RESPONSE 1/AUXIN SIGNALING F-BOX (TIR1/AFB) receptor proteins, AUXIN/IAA (AUX/IAA) repressor proteins and AUXIN RESPONSE FACTOR (ARF) transcriptional factors [9,10]. In this pathway, auxin promotes the ubiquitination and degradation of Aux/IAA proteins by enhancing the interaction between AUX/IAA and TIR1/AFB receptors, and ARF is derepressed from the AUXIN/IAA-ARF complex and then regulates the expression of auxin-responsive gene [11,12]. Interactions of AUXIN/IAA-ARF regulate lateral root initiation and development in many plants, including monocots and dicots [13,14,15,16,17]. In apple, MdIAA27 directly interacts with MdARF8, MdARF26 and MdARF27 to regulate the transcription of *MdSAUR76* and *MdLBD16* and promote the development of adventitious roots (ARs) [18]. The *SAUR* (Small Auxin-Up RNA) gene family is the biggest family among early auxin response genes that rapidly respond to auxin [19]. The role of *SAUR* genes in root development has been well documented. A recent study revealed that the transcription of *PagSAUR36* was regulated by PagWOX11/12a, and overexpression of *PagSAUR36* substantially increased the formation and growth of adventitious roots in poplar [20].

In citrus, the *AUX/IAA* family has been characterized, along with some members related to fruitlet abscissions and somatic embryogenesis [21,22]. However, the function of this gene family in the root development of citrus has not been documented, and the members of ARFs that are able to interact with IAA to fit into the IAA-ARF module are yet to be identified. Our previous study showed that in citrus, PtrSAUR32 acted downstream of the auxin response factor PtrARF8 to regulate root growth by interacting with PtrPP2C.Ds [23]. In this study, we revealed that PtrARF8 interacted with PtrIAA12 to form an AUXIN/IAA-ARF complex to regulate the transcription of the *PtrSAUR17* regulator and affect the growth of roots in citrus (*Poncirus trifoliata*, hereafter referred to as citrus).

## 2. Results

### 2.1. Identification of the Members of PtrAUX/IAAs Involved in Root Development in Citrus

**Figure 1:**
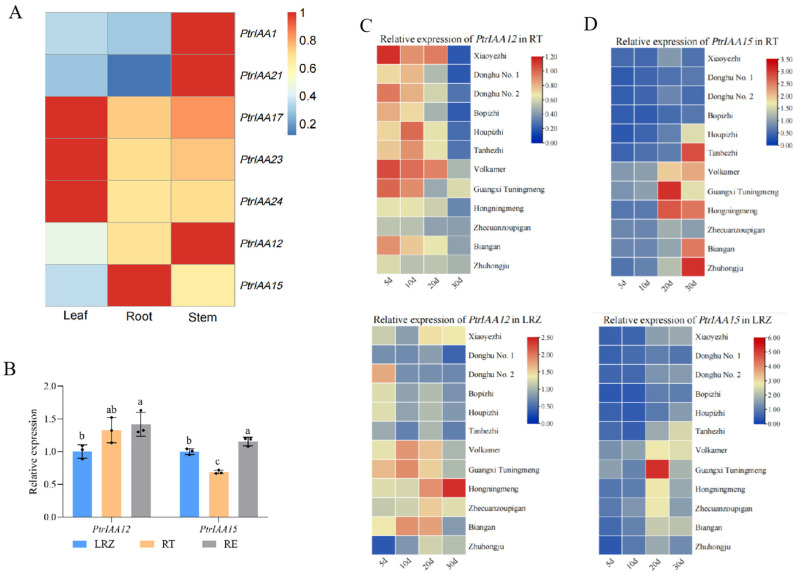
Identification of root development-related *PtrAUX/IAAs* in citrus. (**A**) Expression patterns of *PtrAUX/IAAs* in root, stem and leaf. The data underwent logarithmic transformation. (**B**) Expression analysis of *PtrIAA15* and *PtrIAA12* in different root zones. RT (Root Tip): meristem/elongation zone of roots; RE (Elongation of Roots): root elongation/differentiation and lateral root initiation zone; LRZ: lateral root growth zone. The error bars represent SD. Letters (a–c) represent significant difference, *p* < 0.05. (**C**) Expression patterns of *PtrIAA12* in RT zones and LRZs. (**D**) Expression patterns of *PtrIAA15* in RT zones and LRZs.

The *AUX/IAA* gene family plays a crucial role in root development [24]. In this study, we identified the members of the *AUX/IAA* family and their function in root growth and development in citrus. Thirty-five *IAAs* were retrieved from the trifoliate orange (*P.* *trifoliata*) genome and termed *PtrIAAs.* Among them, seven homologs of root growth-related *AtIAA*s [25,26,27,28,29,30,31,32] were selected to determine their relationship with root growth in citrus. Seven *PtrIAA*s demonstrated different levels of expression in different tissues of citrus plants. *PtrIAA15* was dominantly expressed in the roots, while *PtrIAA12* also exhibited a higher level of expression in roots (Figure 1A). Further investigation revealed that *PtrIAA15* and *PtrIAA12* were expressed differently in different zones of roots. Figure 1B showed that *PtrIAA12* was expressed at a higher level in the RE (Elongation of Roots, the root elongation/differentiation and lateral root initiation zone) and RT (Root Tip, the meristem/elongation zone of root), while *PtrIAA15* had the highest expression in the RE, followed by the LRZ (lateral root growth zone) and RT. In the roots of seedlings of 12 citrus varieties, *PtrIAA12* was expressed during the early period and late period of root growth in the RT and LRZ, respectively, while *PtrIAA15* was mainly expressed in the RT during the late period of root growth (Figure 1C,D). Since *PtrIAA12* showed high activity in the RT from initial root growth across most of the tested citrus varieties, it was considered a major candidate gene for further functional characterization in this study.

### 2.2. PtrIAA12 Participates in the Regulation of Root Growth in Citrus via Interacting with PtrARFs

**Figure 2:**
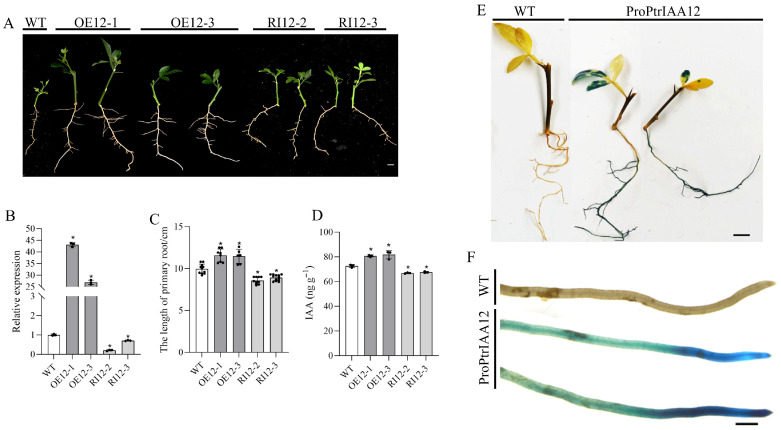
Functional analysis of *PtrIAA12* in transgenic citrus. (**A**) Root phenotype of *PtrIAA12* transgenic plants. Bar = 1 cm. (**B**) The expression of *PtrIAA12* in the roots of transgenic plants. (**C**) Statistical analysis of primary root length. (**D**) IAA contents of roots. (**E**) Organizational analysis of ProPtrIAA12::GUS transgenic plants. Bar = 1 cm. (**F**) GUS staining on root tips. Bar = 1 mm. The error bars represent SD. * *p*  <  0.05.

*PtrIAA12* overexpression and silenced transgenic *P. trifoliata* plants were generated for clarifying the function of *PtrIAA12* in citrus root development. Two lines of *PtrIAA12*-overexpressed plants (OE12-1 and OE12-3; we did not obtain the cuttings to generate seedlings of OE12-2 because of the slow growth of plants after grafting) and two lines of *PtrIAA12*-RNAi plants (RI12-2 and RI12-3), with substantial changes in *PtrIAA12* expression compared to the wild type (WT) (Figure S2), were chosen for generating seedlings from the cuttings for the study. The expression of *PtrIAA12* was significantly upregulated and downregulated in the roots of *PtrIAA12*-overexpressed and RNAi plants, respectively (Figure 2B). Compared to WT plants, the growth of the primary roots of *PtrIAA12*-overexpressed plants was significantly promoted, and that of RNAi plants was significantly inhibited (Figure 2A,C), indicating that *PtrIAA12* is an important regulator in modulating the growth of roots in citrus. We further assessed IAA concentration in the roots of transgenic plants. Compared with WT plants, a significantly higher and lower concentration of IAA was observed in the roots of *PtrIAA12*-overexpressed and RNAi plants (*p* < 0.05), respectively (Figure 2D).

**Figure 3:**
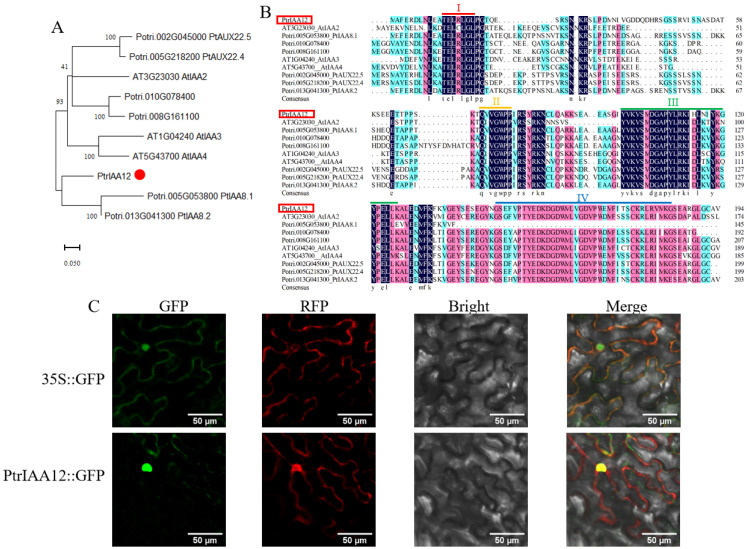
Phylogenic analysis and subcellular localization of PtrIAA12. (**A**) The phylogenetic relationship of *PtrIAA12* with its related putative orthologs from *Arabidopsis* and poplar. *PtrIAA12* was is indicated with a red dot. Blue black background: the similarity of amino acid sequences is 100%; pink: >80% similarity; blue: >50% similarity. (**B**) Multiple sequence alignment of PtrIAA12. Conserved domain I, domain II, domain III and domain IV are underlined. (**C**) Subcellular localization of PtrIAA12.

Bioinformatic analysis revealed that the full-length CDS of *PtrIAA12* is 585 bp, which encodes a putative protein comprising 194 amino acid residues. Phylogenetic analysis indicated that PtrIAA12 shared high similarity with PtIAA8.1 and PtIAA8.2 of poplar, followed by AtIAA3 and AtIAA4 of *Arabidopsis* (Figure 3A). Multiple alignment analysis showed that PtrIAA12 is a canonical Aux/IAA family member, containing four conserved domains (Figure 3B). The subcellular location of PtrIAA12 was verified by co-expression of 35S::PtrIAA12-GFP and the marker with RFP in *N. benthamiana* leaves. The fluorescence of 35S::GFP was observed throughout the cell, while the strong fluorescence signal of 35S::PtrIAA12-GFP was present in the nucleus; a weak fluorescence signal also existed on the membrane, suggesting that PtrIAA12 was mainly located in nucleus (Figure 3C). Presumably, *PtrIAA12* would function as other Aux/IAA paralogs in auxin signal. Tissue-specific expression of *PtrIAA12* was conducted by generating transgenic plants with *GUS* reporter genes driven by *PtrIAA12* promoters (Figure S1). Histochemical staining revealed that *PtrIAA12* was expressed in leaves and roots (Figure 2E), with strong GUS activity in the apical meristem (Figure 2F). It was difficult to investigate the expression of GUS in the stem, due to the intensive brown color of the background and the high optical density of the lignified cell walls of vascular tissues in the stem.

**Figure 4:**
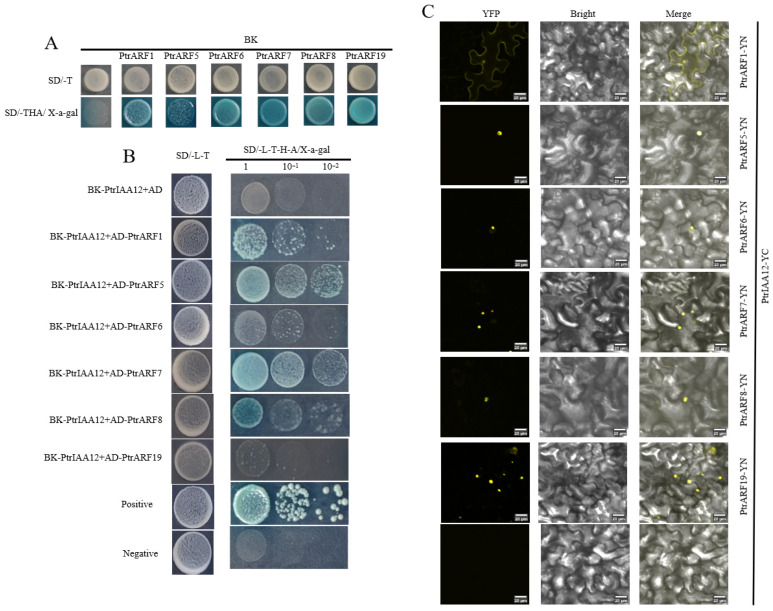
Interactions between PtrIAA12 and PtrARFs. (**A**) Transactivation activity analysis of PtrARFs. (**B**) Interaction assay between PtrIAA12 and PtrARFs via yeast two-hybrid system. SD/-L-T, SD medium without Leu and Trp; SD/-L-T-H-A/X-a-gal, SD medium without Leu, Trp, His and Ade but containing X-α-Gal (40 µg/mL). Positive: pGADT7-T and pGBKT7-53; negative: pGADT7-T and pGBKT7-Lam. (**C**) BiFC assay for PtrIAA12 and PtrARFs. Bars = 20 μm.

In *Arabidopsis*, Aux/IAA proteins repress auxin-inducible genes by inhabiting auxin response transcription factors (ARFs) [33]. The *ARF* gene family has not been reported in *P. trifoliata*. In this study, we identified six *ARF* genes (termed *PtrARF1*, *PtrARF5*, *PtrARF6*, *PtrARF7*, *PtrARF8* and *PtrARF19*) in the *P. trifoliata* genome, which are homologous genes of *CiARFs* in *C. clementina,* as reported by Xie et al. [21]. Those six *CiARFs* were homologs of *Arabidopsis A-ARFs*. The six PtrARFs were fused with the Gal4 DNA-binding domain (BK) to verify their activities in yeast cells. As illustrated in Figure 4A, yeast cells with BK-PtrARFs were viable on SD/-T medium and produced a blue colony on SD/-THA/X-α-Gal medium, suggesting that PtrARF1, PtrARF5, PtrARF6, PtrARF7, PtrARF8 and PtrARF19 could activate the expression of *His3*, *Ade2* and *Mel1* reporter genes in yeast. This indicates that all six PtrARFs are transcriptionally self-activable.

The results of the Y2H assay showed that PtrIAA12 interacted differently with six PtrARFs, showing strong interactions with PtrARF1, PtrARF5, PtrARF7 and PtrARF8 and weak interactions with PtrARF6 and PtrARF19 (Figure 4B). In vivo interactions of PtrIAA12 and PtrARFs were confirmed by a BiFC assay. A strong YFP signal was observed on the *N. benthamiana* leaves co-transformed with the vectors of PtrIAA12 fused to CYFP, and PtrARFs fused to NYFP (Figure 4C).

Six *PtrARFs* were expressed in all three tissues at different levels. Among them, *PtrARF7* and *PtrARF19* demonstrated significantly higher levels of expression in roots. The expressions of six *PtrARFs* also varied in different zones of roots, of which four *PtrARFs* (*PtrARF6*, *PtrARF7*, *PtrARF8* and *PtrARF19*) showed higher levels of expression in the RT. The above results suggest that *PtrARFs* may be involved in the regulation of the root morphogenesis in citrus (Figure S3).

### 2.3. PtrARF8 Regulates the Expression of PtrSAUR17

**Figure 5:**
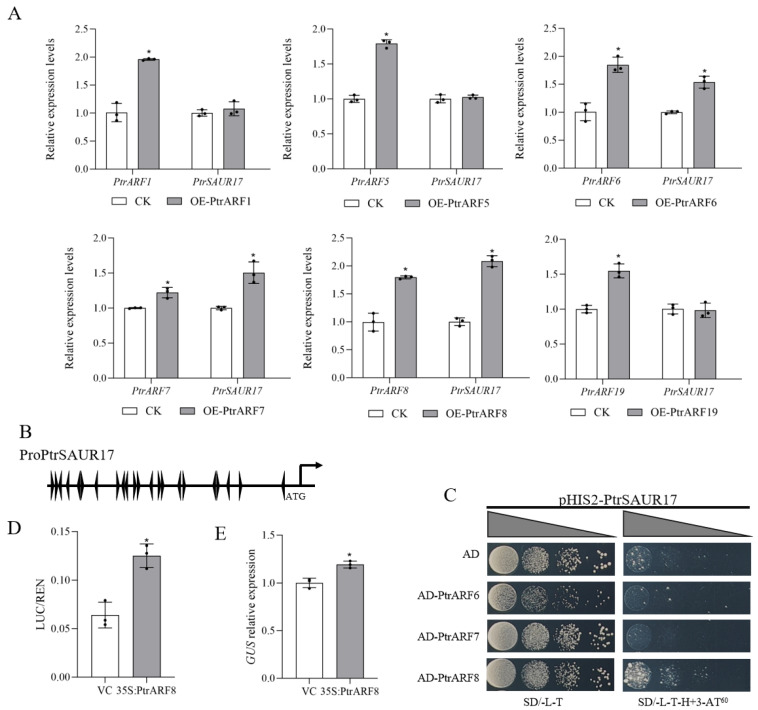
Interactions between PtrARFs and *PtrSAUR17*. (**A**) Transcript levels of PtrSAUR17 in citrus leaves infiltrated with PtrARFs. (**B**) Auxin-responsive elements (AuxREs) in the promotor of *PtrSAUR17*. (**C**) Y1H assays of PtrARFs and PtrSAUR17 promoter. SD/-L-T: SD medium without Leu and Trp; SD/-L-T-H+3-AT: SD medium without Leu, Trp and His but containing 3-AT. (**D**) Transient transactivation of dual-luciferase reporter assay. LUC, firefly luciferase, REN, Renillia luciferase. VC, vector control. (**E**) Transient transactivation assay using GUS reporter gene. VC, vector control. The error bars represent SD. Asterisk indicates significant difference, * *p*  <  0.05.

In *Arabidopsis*, *SAUR15* is necessary for auxin-mediated lateral root and adventitious root formation under the regulation of some ARFs [34]. Our previous study also revealed that in citrus, *PtrSAUR17* and *PtrSAUR32* were highly expressed in roots, and *PtrSAUR32* was transcriptionally regulated by PtrARFs to affect the growth of the roots [23]. In this study, transient over-expression of *PtrARF6*, *PtrARF7* and *PtrARF8* in citrus leaves significantly upregulated the expression of *PtrSAUR17* (Figure 5A), suggesting that *PtrARF6*, *PtrARF7* and *PtrARF8* might regulate the expression of *PtrSAUR17*.

The *PtrSAUR17* promoter contains several AuxRE elements (Figure 5B). The yeast one-hybrid (Y1H) assay analysis showed that yeast cells containing AD-PtrARF8 and pHIS2-PtrSAUR17 promoters grew well on the deficiency medium with 60 mM 3-AT, while the yeast cells harboring the pHIS2-PtrSAUR17 promoter together with AD (control), AD-PtrARF6 and AD-PtrARF7 grew poorly, indicating that among the three PtrARFs, only PtrARF8 can physically bind to the *PtrSAUR17* promoter in vitro (Figure 5C).

The interaction between PtrARF8 and the promoter of PtrSAUR17 was further verified by a dual-luciferase (LUC) assay in *N. benthamiana* leaves with a LUC reporter gene driven by a promoter of *PtrSAUR17*. The results showed that the LUC/REN ratio of *PtrSAUR17* promoter co-expression with PtrARF8 was significantly higher than that of *PtrSAUR17* promoter co-expression with the VC (vector control) (Figure 5D). A GUS assay was performed to examine the effect of PtrARF8 on *PtrSAUR17* transcription. The relative *GUS* expression levels in *N. benthamiana* leaves co-transformed with the *PtrSAUR17* promoter and PtrARF8 were significantly greater than that co-transformed with the *PtrSAUR17* promoter and the VC (Figure 5E). These results suggest that PtrARF8 directly binds to the promoter of *PtrSAUR17* and enhances its expression.

### 2.4. PtrSAUR17 Promotes Root Growth in Citrus

**Figure 6:**
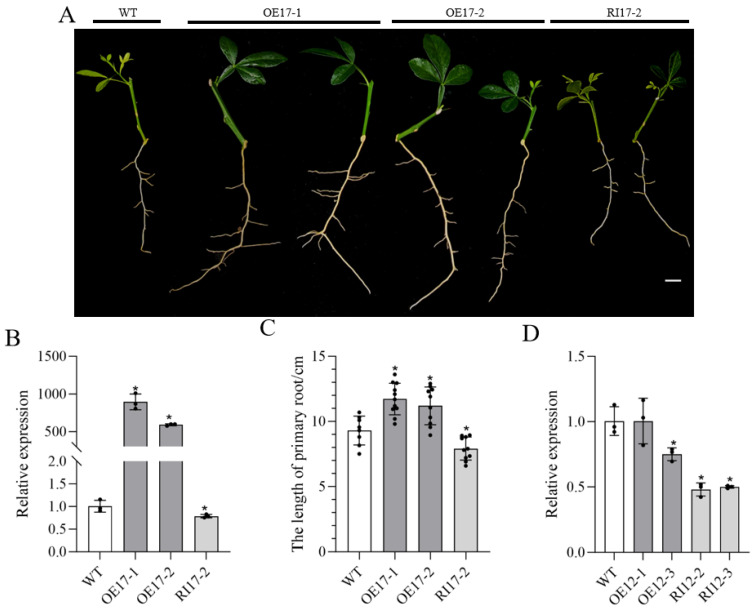
Functional analysis of *PtrSAUR17* in citrus roots. (**A**) Root phenotype of *PtrSAUR17* transgenic plants. Bar= 1 cm; (**B**) The expression of *PtrSAUR17* in the roots of transgenic plants. (**C**) Statistical analysis of primary root length. (**D**) The expression of *PtrSAUR17* in the roots of *PtrIAA12* transgenic plants. The error bars represent SD. Asterisk indicates significant differences between transgenic plants and WT, * *p*  <  0.05.

*PtrSAUR17* transgenic citrus plants were developed to generate the seedlings from cuttings for verifying the function of *PtrSAUR17* in the root development of citrus. Up- and downregulated expression of *PtrSAUR17* in transgenic plants remarkably affected the growth of roots (Figure 6A). Compared with the WT, the expression of *PtrSAUR17* was significantly upregulated and downregulated in the roots of *PtrSAUR17*-overexpressed and -silenced lines, respectively (Figure 6B). At the same time, the growth of primary roots significantly increased, by 26% and 20.4%, in the OE17-1 and OE17-2 line (*p*  <  0.05), respectively, and decreased, by 14.9%, in the RI17-2 line (*p*  <  0.05) (Figure 6C). These results indicate that the *PtrSAUR17* plays an important role in the growth and development of citrus roots. In the roots of *PtrIAA12*-overexpressed plants, the expression of *PtrSAUR17* was significantly higher than that in *PtrIAA12*-silenced plants. However, compared to both overexpressed and silenced plants, the expression of *PtrSAUR17* in wild-type plants was higher (Figure 6D).

## 3. Discussion

Auxin is central to nearly every facet of root development [5]. AUX/IAA and ARFs are important components of auxin signals. Generally, AUX/IAA is a functional protein, binding to ARFs [35]. Auxin leads to the release of ARFs from the AUX/IAA-ARF heterodimer by promoting the ubiquitination and degradation of Aux/IAA proteins; ARF then binds to auxin response elements (AuxREs) and regulates the transcription of downstream genes [36,37]. Studies have shown that ARF proteins bind to AuxREs to regulate auxin-mediated growth and development [38,39]. Citrus is one of most important fruit trees that depends on grafting for propagation. The root system of rootstocks plays a fundamental role in the growth of citrus plants. The development and growth of roots are the key components to determine the structure and function of the root system. However, molecular mechanisms regulating citrus root growth and development have not been well documented. In this study, we identified that a key component of auxin signaling, PtrIAA12, played an important role in citrus root growth by interacting with PtrARFs to regulate the expression of *PtrSAUR17*. Our findings reveal a potential target for improving the root system of citrus rootstocks.

**Figure 7:**
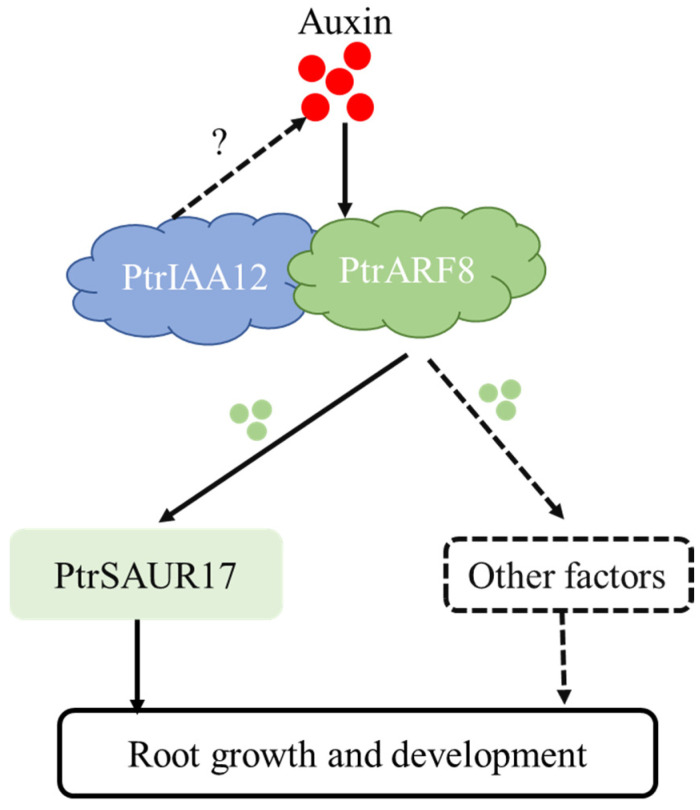
A model for *PtrIAA12*-regulated root growth and development in *P. trifoliata*.

As an important transcription factor in auxin signaling transduction, Aux/IAA was crucial in regulating root development [14,17,40]. Some studies have indicated that Aux/IAA also plays an important role in abiotic stress tolerance [41,42,43,44]. In this study, one of the members of citrus *AUX/IAA* genes, *PtrIAA12,* was identified as a key gene regulating the growth of roots in citrus. PtrIAA12 could interact with several PtrARF members to form the AUX/IAA-ARF complex, as reported in *Arabidopsis* [33,45]. In *Arabidopsis*, activator AtARFs have been reported to be involved in root organogenesis [46,47,48,49,50], and multiple AtAUX/IAA-AtARF modules regulate root growth and development [14]. Overexpression and silence of *PtrIAA12* in citrus resulted in enhanced and suppressed growth of the root, respectively, indicating that the PtrIAA12-PtrARF complex in citrus is also involved in the regulation of root growth and development. It may also affect the abiotic stress tolerance of roots, which is important for rootstocks. However, this requires further study to clarify whether *PtrIAA12* could improve the tolerance of citrus to abiotic stress. The study further revealed that PtrARF8 interacted with *PtrSAUR17* as a transcriptional factor to regulate its expression, therefore regulating the growth and development of roots in citrus. In *Arabidopsis*, *SHY2/IAA3* negatively controlled root meristem size and growth [25]. However, *PtrIAA12*, the homolog of *SHY/IAA3*, was found to positively regulate citrus root growth in this study, which is in agreement with the findings for *MdIAA27* and *ZmIAA5* [17,18]. Similarly, higher and lower levels of auxin were also present in the roots of PtrIAA12-overexpressed and -silenced plants, respectively. It has been documented that auxin promotes the ubiquitination and degradation of Aux/IAA proteins, helping release ARF proteins from the AUXIN/IAA-ARF complex and then regulating the expression of auxin-responsive genes [11,12]. Here, promoting root growth by *PtrIAA12* could have been due to the higher and lower level of auxin in the roots of *PtrIAA12*-overexpressed and -silenced plants, which released more and less PtrARF8 proteins from the PtrIAA12-PtrARF8 complex to increase and decrease the growth of primary roots in transgenic citrus plants by regulating the expression *PtrSAUR17*. We found that expression of *PtrSAUR17* was significantly higher in *PtrIAA12*-overexpressed and plants than in silenced plants. But expression of *PtrSAUR17* was higher in WT plants than in overexpressed plants. The cause of this phenomenon needs to be identified in future work. Based on above results, we speculate that PtrARF8 released by auxin may simultaneously regulate other genes, which may promote root growth and development and cooperate with *PtrSAUR17* to regulate root development (Figure 7).

The *SAUR* genes form a plant-specific gene family and play positive roles in root development and growth, shown in several recent studies [20,34,51,52]. It was noteworthy that SAUR interacted with PP2C.D and then activated PM H^+^-ATPases to promote cell expansion and drive the growth of organs [53,54,55]. Root cell elongation is the primary driving force of root growth. This study clarified that the PtrIAA12-PtrARF8 complex mediates the growth and development of roots in citrus through regulating the transcription of *PtrSAUR17*. Whether *PtrSAUR17*-regulated root growth is the consequence of cell expansion, activated by the interaction of PtrSARU17 with PP2C.Ds, needs to be proved in a further study.

## 4. Materials and Methods

### 4.1. Plant Materials

The listed citrus germplasm resources used as plant materials in this study were provided by the National Citrus Germplasm Repository (Chongqing, China)—*Poncirus trifoliata*: ‘Donghaizhi’, ‘Tanhezhi’, ‘Donghu No. 1’, ‘Donghu No. 2’, ‘Xiaoyezhi’, ‘Houpizhi’ and ‘Bopizhi’; *Citrus reticulata*: ‘Biangan’, ‘Zhuhongju’ and ‘Zhecuanzoupigan’.; *C. limon*: ‘Hongningmeng’; *C. limonia:* ‘Guangxi Tuningmeng’; and *C. volkameriana*: ‘Volkamer’. ‘Donghaizhi’ was used for investigation of *PtrAUX/IAA* expressions in leaf, root and stem and different zones of root, as well as the generation of transgenic citrus. All the other varieties were used for investigation of gene expressions at different growth stages of roots. Plants of *Nicotiana benthamiana* were used for subcellular localization analysis and bi-molecular fluorescence complementation (BiFC) analysis.

The samples of different tissues and organs, as well as different zones of roots, were collected from young seedlings cultured in a growth room under a temperature of 26 °C. The different zones of roots were termed the RT (Root tip), for the meristematic/elongation zone; the RE (Elongation of Roots), for the root elongation/differentiation and lateral root initiation zone; and the LRZ, for the lateral root growth zone, according to Zhang et al. [56] and Hwang et al. [57].

### 4.2. Gene Expression Analysis

To analyze the expression of genes at different developmental stages, samples of RT zones and LRZs were collected at 5, 10, 20 and 30 days after citrus plants were cultured at 26 °C in a temperature-controlled environment. Genomic DNA and total RNA were extracted according to the protocol of the CTAB Plant DNA Extraction Kit (DL114) and instruction of the EASYspin Plus Plant RNA Kit (RN38, Aidlab, Beijing, China, respectively, from citrus and tobacco plants. The single-stranded cDNA was synthesized from total RNA by using the RevertAid™ Master Mix (M16325, Thermo Scientific, Waltham, MA, USA). RT-qPCR was performed using NovoStart^®^SYBR qPCR SuperMix plus (E096-01A, novoprotein, Beijing, China). The relative expression of genes was calculated with the 2^−ΔΔCt^ method [58]. *PtrActin* was used as the reference gene. Three replicates were used to analyze each sample. The primer sequences used for RT-qPCR are listed in Supplementary Table S1.

### 4.3. Phylogenetic Analysis and Multiple Sequence Alignments

The theoretical pI and molecular weight of PtrIAA12 was predicted using the ExPASy website (https://www.expasy.org/, accessed on 10 September 2025). The Phytozome database (https://phytozome-next.jgi.doe.gov/, accessed on 10 September 2025) was used to acquire the sequences of PtrIAA12 and its homologous proteins. The neighbor-joining phylogenetic tree was created utilizing MEGA 11 software, with 1000 bootstrap replicates. The DNAMAN9 software was used to analyze the amino acid sequences between PtrIAA12 and other proteins.

### 4.4. Subcellular Localization Analysis

The recombinant vector harboring Cam35S: PtrIAA12-GFP and control vector Cam35S-GFP were transformed into EHA105 competent cells. *Agrobacterium* cells containing the above vectors were co-transformed with a marker (RFP) into the leaves of 4 week-old tobacco via an *Agrobacterium*-mediated method, described by Yuan and Xu [59]. Two days after infiltration, the confocal scanning microscope FV3000 (Olympus, Tokyo, Japan) was used to observe fluorescence signals. The primer sequences used for vector construction are listed in Supplementary Table S2.

### 4.5. Stable Transformation in Citrus

The vector pFGC5941MDB3F-GN was used as the plant expression vector in this study. The full-length CDS and a specific DNA fragment of *PtrIAA12* and *PtrSAUR17* were inserted into the plant expression vector to generate overexpression and RNA interference (RNAi) lines. The 2500 bp promoter of *PtrIAA12* was fused into p1300GNGM-GUS vector to analyze the activity of the *PtrIAA12* promoter. *Agrobacterium* cells harboring the above recombinant plasmids were transferred into trifoliate orange ‘Donghaizhi’ to generate transgenic citrus plants, as described by Zhang et al. [60].

Buds of transgenic plants were grafted on trifoliate rootstocks for generating shoots for cuttings. Shoot segments (3–5 cm) were cut from transgenic and wild-type (WT) plants about 3 months after grafting and cultivated in vermiculite in a culture room with a temperature of 26 °C. Seedlings with developed roots were obtained 30 days after culturing, and 6–12 seedlings were used for study.

### 4.6. GUS Staining

The seedlings used for histochemical β-glucuronidase (GUS) staining analysis were generated from the cuttings of wild-type (WT) plants and the transgenic plants of the *PtrIAA12* promoter. The seedlings were incubated for 12 h at a temperature of 37 °C in GUS dye solution. After incubation, the samples were decolorized several times with 70% ethanol.

### 4.7. Auxin Analysis

The concentration of IAA was measured according to the instruction of a Plant IAA ELISA Kit (JW.PL1382, GIVEI, Shanghai, China).

### 4.8. Transactivation Analysis in Yeast

The coding sequences of *PtrARFs* were inserted into the pGBKT7 vector, and transferred into the Y2HGold (CC309, Coolaber, Beijing, China) yeast strain according to the protocol. The yeast cells were cultured on SD/-T and SD/-THA/X-α-gal plates at a temperature of 28 °C for 2–3 days.

### 4.9. Y2H and BiFC Assay

For the yeast two-hybrid (Y2H) assay, the full-length sequences of *PtrIAA12* and *PtrARFs* were inserted into yeast expression vectors pGBKT7 and pGADT7, respectively. The recombinant plasmids pGBKT7-PtrIAA12 and pGADT7-PtrARFs were co-transformed into yeast cells according to the protocol of the Y2HGold Chemically Competent Cell (CC309, Coolaber, Beijing, China). The positive colonies growing on SD/-L-T medium were transferred to the plate of SD/-L-T-H-A/X-α-gal for observation of the growth of yeasts.

For the bimolecular fluorescence complementation (BiFC) assay, the CDS of *PtrIAA12* and *PtrARFs* was cloned into pCV-cYFP and pCV-nYFP, respectively. The *Agrobacterium tumefaciens* strain EHA105 was used to mediate the transient expression of the gene in tobacco leaves, according to Yuan and Xu [59]. Two days after co-infiltration, the leaves were used to observe fluorescence signals.

### 4.10. Transient Overexpression of PtrARF Genes in Citrus Leaves

The coding sequences of *PtrARFs* were ligated into the pFGC5941MDB3F-GN vector to generate overexpressed plasmids, and then transformed into the EHA105 strain. The *Agrobacterium* cells with PtrARF-pFGC5941MDB3F-GN plasmids were collected by using an infiltration buffer, containing 10 mM MES, 10 mM MgCl_2_ and 150 μM AS. After 2–5 days of infiltration, the infiltrated leaves were collected to analyze gene expressions.

### 4.11. Y1H and Transient Transactivation Assay

A yeast one-hybrid (Y1H) assay was used to examine the ability of PtrARFs to bind to the *PtrSAUR17* promoter. The coding regions of *PtrARFs* were cloned into the pGADT7 vector to generate AD-prey vectors, and a 2500 bp promoter of *PtrSAUR17* was inserted into the pHIS2 vector pHIS2-bait. Different combinations were co-transformed into Y187 (CC306, Coolaber, Beijing, China) competent cells and were cultured on SD/-L-T plates. The yeast clones were dissolved, diluted with a series of concentrations and cultured on SD/-L-T and SD/-L-T-H/3-AT.

Transient transactivation assay was performed to further detect the regulatory effect of PtrARFs on the promoter of *PtrSAUR17*. The coding region of *PtrARF8* and a fragment of the *PtrSAUR17* promoter were individually inserted into pGreen II 62-SK and pGreenII 0800-LUC vectors to generate an effector construct and reporter construct. These constructs were transformed into the *Agrobacterium* strain GV3101 (AC1002S, WEIDI, Shanghai, China) with the helper plasmid pSoup, respectively, and then co-transformed into tobacco leaves to measure the LUC and REN luminescent signals using the Dual-Luciferase Reporter Gene Assay Kit (11402ES60, Yeasen, Shanghai, China). Next, the *PtrSAUR17* promoter fragment was amplified and ligated into the p1300GNGM-GUS vector to drive *GUS* gene expression. The above overexpressed plasmid of PtrARF8-pFGC5941MDB3F-GN was used as an effector in a transient expression assay of GUS. After co-injection into tobacco leaves for 2 days, RNA was extracted to synthesize cDNA and measure the expression of *GUS*.

### 4.12. Accession Numbers

The sequence data in this study were deposited in the Phytozome (https://phytozome-next.jgi.doe.gov/, accessed on 10 September 2025) databases: *PtrIAA17* (Ptrif.0001s0445), *PtrIAA23* (Ptrif.0005s0101), *PtrIAA24* (Ptrif.0003s4932), *PtrIAA15* (Ptrif.0009s0907), *PtrIAA12* (Ptrif.0005s1113), *PtrIAA1* (Ptrif.0009s0669), *PtrIAA21* (Ptrif.0001s2346), *PtrARF1* (Ptrif.0002s2486), *PtrARF6* (Ptrif.0002s0903), *PtrARF8* (Ptrif.0006s1621), *PtrARF19* (Ptrif.0001s2887), *PtrARF7* (Ptrif.0001s2230), *PtrARF5* (Ptrif.0005s2742), *PtrSAUR17* (Ptrif.0004s1277), and *PtrActin* (Ptrif.0007s2253).

### 4.13. Statistical Analysis

GraphPad Prism 10 was used to analyze and visualize the data. Analyses of statistical differences were performed by one-way ANOVA or *t*-tests. The data were shown as “mean ± standard deviation (SD)” based on at least three biological replicates.

## Data Availability

Data are contained within the article and Supplementary Materials.

## Supplementary Materials

The following supporting information can be downloaded at: https://www.mdpi.com/article/10.3390/plants14182875/s1, Figure S1: ProPtrIAA12::GUS fragment in transgenic citrus plants; Figure S2: Confirmation of PtrIAA12 transgenic citrus plants; Figure S3: The expression profiles of PtrARFs; Figure S4: Identification of PtrSAUR17 transgenic citrus; Table S1: Primers used for qRT-PCR analysis; Table S2: Primers used for vector construction.
