# Supplementary material for: PtrIAA12-PtrARF8 Complex Regulates the Expression of PtrSAUR17 to Control the Growth of Roots in Poncirus trifoliata
Source: Plants (Basel). 2025 Sep 16;14(18):2875. doi: 10.3390/plants14182875 (PMC12473356; doi:10.3390/plants14182875)
Supplement: Supplementary file 1 [file plants-14-02875-s001.zip › Supplementary Materials.pdf]

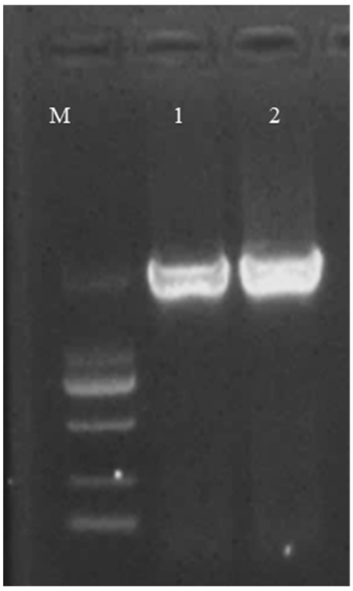

Fig. S1 ProPtrIAA12::GUS fragment in transgenic citrus plants

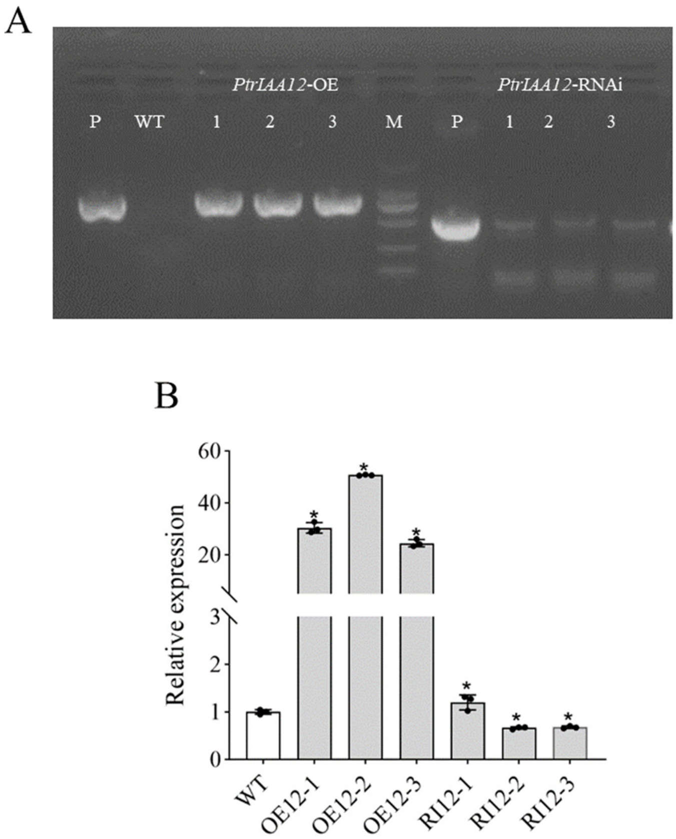

Fig. S2 Confirmation of *PtrIAA12* transgenic citrus plants. A PCR validation of *PtrIAA12* overexpression (OE) and RNA interference (RNAi) transgenic plants. **B** The expression level of *PtrIAA12* in overexpression and RNAi plants. Asterisk indicates significant difference between transgenic plants and WT, **P* < 0.05.

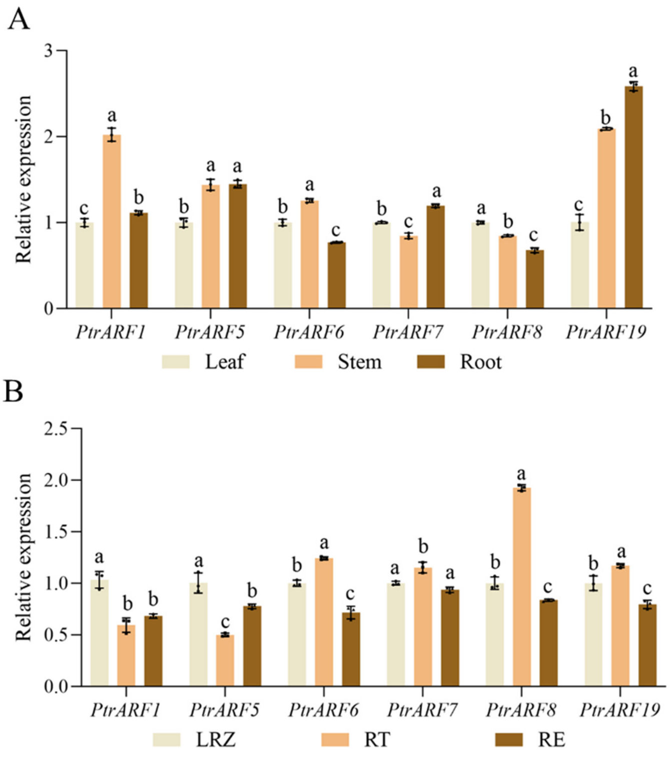

Fig. S3 The expression profiles of *PtrARFs*. A Expression of *PtrARFs* in root, stem and leaf. **B** Expression of *PtrARFs* in different zones of citrus root. Letters (a-c) represent significant difference, $p < 0.05$.

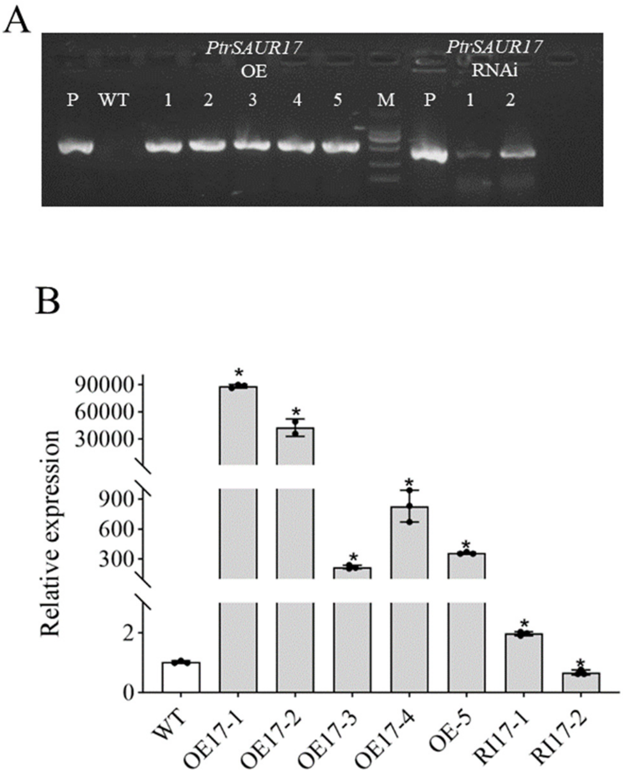

Fig. S4 Identification of *PtrSAUR17* transgenic citrus. A PCR validation of *PtrSAUR17* overexpression (OE) and RNA interference (RNAi) transgenic plants. **B** The expression level of *PtrSAUR17* in the leaves of overexpression and RNAi plants. Asterisk indicates significant difference between transgenic plants and WT, $*P < 0.05$.

Table S1 Primers used for qRT-PCR analysis

Gene	Forward primer (5′ to 3′)	Reverse primer (5′ to 3′)
PtrIAA12	CTCCGCCTAGCAAGACACAA	GCCTCCGATTTCTTCGCTTG
PtrIAA1	GACGGGGCTCCATATCTTCG	CCAAGGAACATCACCCACGA
PtrIAA17	AATTGAGGCTTGCTCCTCCA	CCCTCTTGCTTACCAGCCAC
PtrIAA15	CCCAGCTATCAATTCCATAGGT	ATTCGAAGCCTTTTTACTGAGC
PtrIAA21	CCAACGCATCAGTCTTCATAAG	ACTCGTACAAAATCCTTCCAGT
PtrIAA23	ACCTGCAGGCTATTTGGCAT	TGCTCGATCATCCCCCATTG
PtrIAA24	GAAGGGATCCCAATCGGCAG	TCCAGTCCCCTTCCTTGTCT
PtrSAUR17	AAGAAAGGTTTCCAAGTTTCCG	AGTCCTTGTTGATGATAGCCAA
PtrARF19	GCAAATGGGTTTCTGCCAAGT	AGGGAAGTAAACAACCAGGCT
PtrARF5	TGGAGGATTGGTTATTCGAGCA	TGAGGGAGGAAAACAAGGGG
PtrARF6	ACGTCGGGGTTTAATCAGCA	AGACAACACGGCTTCCAACA
PtrARF8	GTGTCAACAGGGTCATGAAGG	CTGTTCGCTATGCCCCTGAG
PtrARF1	GGCCCTCTGGTTTCTCTTCC	GGGTAGTTAGGAATATGGGCATCC
PtrARF7	GGAGGAGGGGAGAAGAAGACT	TCTTCATAGATGCTGCCACCT
PtrActin	CCGACCGTATGAGCAAGGAAA	TTCCTGTGGACAATGGATGGA

Table S2 Primers used for vector construction

Name	Forward primer (5′ to 3′)	Reverse primer (5′ to 3′)
OE-PtrIAA12	GCCCAATCGATGATTTAAATATGGCATTTGAGAGAGAT	CTCTAGACTCACCTAGGATCCTTAAACAGCACAGCC
OE-PtrARF1	GCCCAATCGATGATTTAAATATGAGGCTCTCTGCAGCT	CTCTAGACTCACCTAGGATCCTCAGAAATCCAGTGA
OE-PtrARF5	GCCCAATCGATGATTTAAATATGGGTTCTGTTGAAGA	CTCTAGACTCACCTAGGATCCTCAAGCACGGCCACC
OE-PtrARF6	GCCCAATCGATGATTTAAATATGAGACTCGCTACGTCG	CTCTAGACTCACCTAGGATCCTCAGTAGTTGATAGAC
OE-PtrARF7	GCCCAATCGATGATTTAAATATGAAGACACCGGCAAA	CTCTAGACTCACCTAGGATCCTTAAGCATTCCCATTA
OE-PtrARF8	GCCCAATCGATGATTTAAATATGAAGCTTTCAACATCA	CTCTAGACTCACCTAGGATCCTCAATATTCAAGCGAA
OE-PtrARF19	GCCCAATCGATGATTTAAATATGAAGCCTCCAGCAAA	CTCTAGACTCACCTAGGATCCTTATCGATTAAATGAG
RNAi-PtrIAA12	GCCCAATCGATGATTTAAATGAAGAAACAACTCCGCC	AAACCTGACGTCATTTAAATCATGACCCTTAGCCTTT
	TCGTCTTTGTAGTCTCTAGAGAAGAAACAACTCCGCC	AATGCATGCTTAGGTGGATCCCATGACCCTTAGCCTT
Sub- PtrIAA12	AGAACACGGGGGACGAGCTCATGGCATTTGAGAGAG	CGACTCTAGAGGATCCTTAAACAGCACAGCCTAGGC
GUS-PtrIAA12	CGACGGCCAGTGCCAAGCTTACGCCACCTAAGTCTCT	GGACTGACCACCCGGGGATCCGGCCTTACTTGTAAT
GUS-PtrSAUR17	GCCAAGCTTGCATGCCTGCAGACAAGGCTCGTCATCA	GGACTGACCACCCGGGGATCCTTCCTTTCAGGAATC
BK-PtrIAA12	TGGCCATGGAGGCCGAATTCATGGCATTTGAGAGAGA	CGCTGCAGGTCGACGGATCCTTAAACAGCACAGCCT
BKPtrARF1	TGGCCATGGAGGCCGAATTCATGAGGCTCTCTGCAGC	CGCTGCAGGTCGACGGATCCTCAGAAATCCAGTGAC
BK-PtrARF5	TGGCCATGGAGGCCGAATTCATGGGTTCTGTTGAAGA	CGCTGCAGGTCGACGGATCCTCAAGCACGGCCACCT
BK-PtrARF6	TGGCCATGGAGGCCGAATTCATGAGACTCGCTACGTC	CGCTGCAGGTCGACGGATCCTCAGTAGTTGATAGAC
BK-PtrARF7	TGGCCATGGAGGCCGAATTCATGAAGACACCGGCAA	CGCTGCAGGTCGACGGATCCTTAAGCATTCCCATTAT
BK-PtrARF8	TGGCCATGGAGGCCGAATTCATGAAGCTTTCAACATC	CGCTGCAGGTCGACGGATCCTCAATATTCAAGCGAA
BK-PtrARF19	TGGCCATGGAGGCCGAATTCATGAAGCCTCCAGCAA	CGCTGCAGGTCGACGGATCCTTATCGATTAAATGAG
AD-PtrARF1	GTACCAGATTACGCTCATATGATGAGGCTCTCTGCAG	ATGCCCACCCGGGTGGAATTCTCAGAAATCCAGTGA
AD-PtrARF5	GTACCAGATTACGCTCATATGATGGGTTCTGTTGAAG	ATGCCCACCCGGGTGGAATTCTCAAGCACGGCCACC
AD-PtrARF6	GTACCAGATTACGCTCATATGATGAGACTCGCTACGTC	ATGCCCACCCGGGTGGAATTCTCAGTAGTTGATAGA
AD-PtrARF7	GTACCAGATTACGCTCATATGATGAAGACACCGGCAA	ATGCCCACCCGGGTGGAATTCTTAAGCATTCCCATTA
AD-PtrARF8	GTACCAGATTACGCTCATATGATGAAGCTTTCAACATC	ATGCCCACCCGGGTGGAATTCTCAATATTCAAGCGA
AD-PtrARF19	GTACCAGATTACGCTCATATGATGAAGCCTCCAGCAA	ATGCCCACCCGGGTGGAATTCTTATCGATTAAATGAG
YC-PtrIAA12	GAGCTGTACAAGTCCGGAGTCGACATGGCATTTGAG	GAATTCGAGCTCGCCTGGGGATCCTTAAACAGCACA
YN-PtrARF1	AACATCGAGGACTCCGGAGTCGACATGAGGCTCTCT	GAATTCGAGCTCGCCTGGGGATCCTCAGAAATCCAG
YN-PtrARF5	AACATCGAGGACTCCGGAGTCGACATGGGTTCTGTT	GAATTCGAGCTCGCCTGGGGATCCTCAAGCACGGCC
YN-PtrARF6	AACATCGAGGACTCCGGAGTCGACATGAGACTCGCT	GAATTCGAGCTCGCCTGGGGATCCTCAGTAGTTGAT
YN-PtrARF7	AACATCGAGGACTCCGGAGTCGACATGAAGACACCG	GAATTCGAGCTCGCCTGGGGATCCTTAAGCATTCCCA
YN-PtrARF8	AACATCGAGGACTCCGGAGTCGACATGAAGCTTTCA	GAATTCGAGCTCGCCTGGGGATCCTCAATATTCAAG
YN-PtrARF19	AACATCGAGGACTCCGGAGTCGACATGAAGCCTCCA	GAATTCGAGCTCGCCTGGGGATCCTTATCGATTAAAT
pHIS2-PtrSAUR17	ACGACTCACTATAGGGCGAATTCTCGTCATCATCTGCT	TTCGCGAACGCGTGAGCTCCTCTCCCCAAATTTATAT
LUC- PtrSAUR17	GGCCCCCCCTCGAGGTCGACACAAGGCTCGTCATCAT	GCTCTAGAACTAGTGGATCCTTCCTTTCAGGAATCTC
SK- PtrARF8	CCCGGGCTGCAGGAATTCATGAAGCTTTCAACATCAG	TCGACGGTATCGATAAGCTTTCAATATTCAAGCGAAC
