# Supplementary figures and images for: PtrIAA12-PtrARF8 Complex Regulates the Expression of PtrSAUR17 to Control the Growth of Roots in Poncirus trifoliata
Source: Plants (Basel). 2025 Sep 16;14(18):2875. doi: 10.3390/plants14182875 (PMC12473356; doi:10.3390/plants14182875)

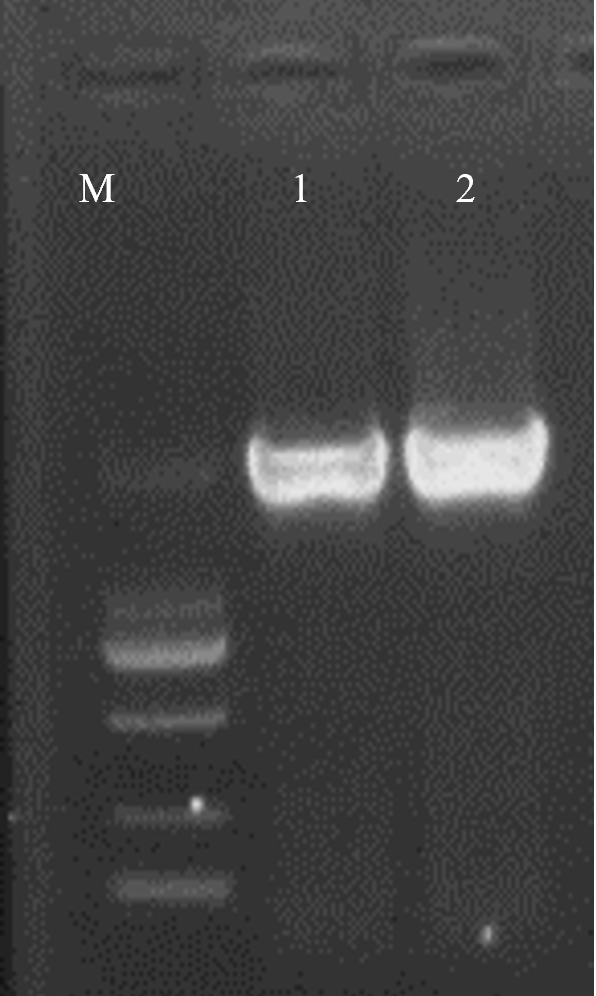

Supplement: Supplementary file 1 [file plants-14-02875-s001.zip › Fig S1.tif]

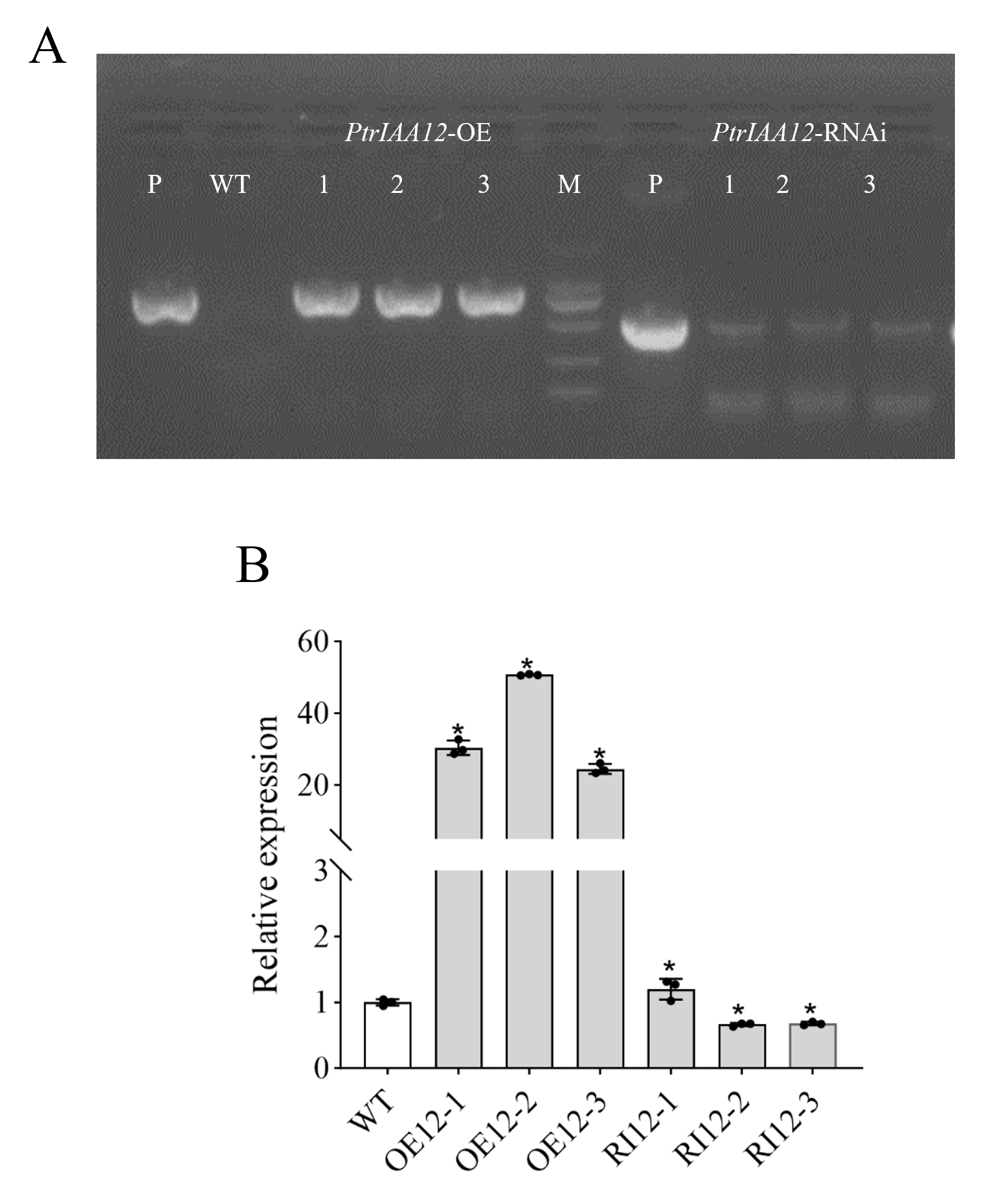

Supplement: Supplementary file 1 [file plants-14-02875-s001.zip › Fig S2.tif]

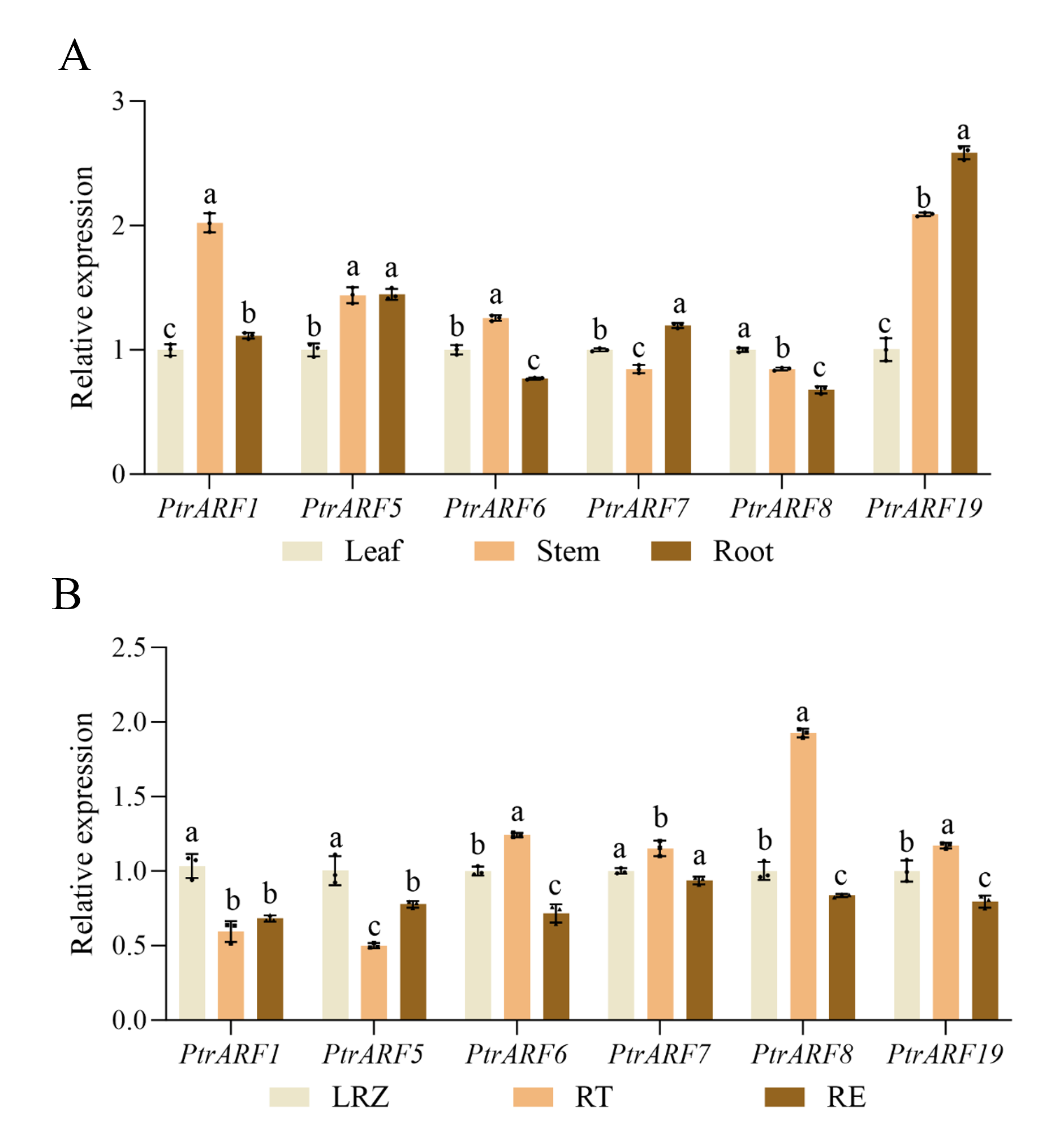

Supplement: Supplementary file 1 [file plants-14-02875-s001.zip › Fig S3.tif]

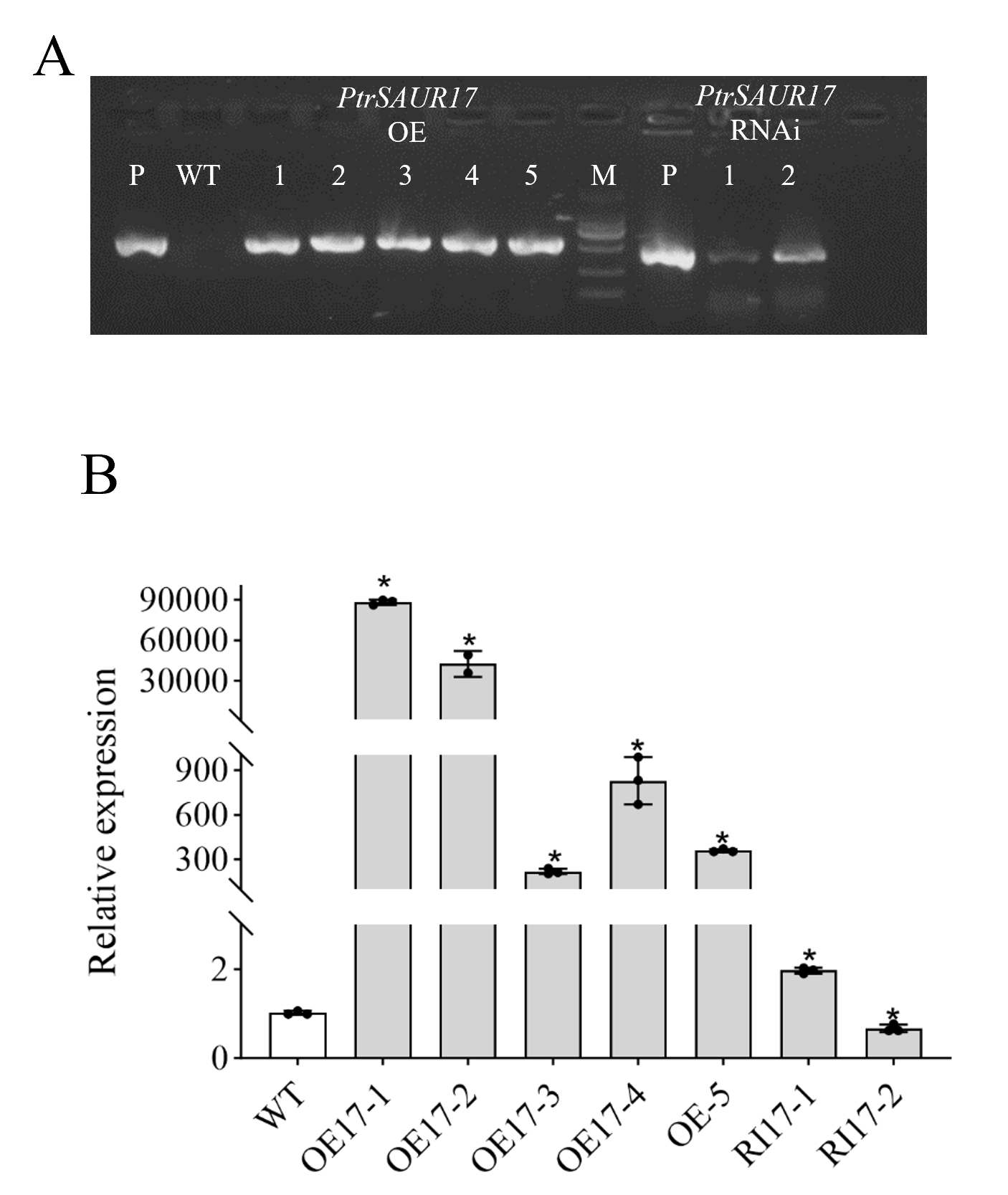

Supplement: Supplementary file 1 [file plants-14-02875-s001.zip › Fig S4.tif]
